# Ecological Relationships of Meso-Scale Distribution in 25 Neotropical Vertebrate Species

**DOI:** 10.1371/journal.pone.0126114

**Published:** 2015-05-04

**Authors:** Lincoln José Michalski, Darren Norris, Tadeu Gomes de Oliveira, Fernanda Michalski

**Affiliations:** 1 Programa de Pós-Graduação em Ecologia, Instituto Nacional de Pesquisas da Amazônia, Manaus, Amazonas, Brazil; 2 Laboratório de Ecologia e Conservação de Vertebrados, Universidade Federal do Amapá, Macapá, Amapá, Brazil; 3 Departamento de Biologia, Universidade Estadual do Maranhão, São Luís, Maranhão, Brazil; 4 Instituto Pró-Carnívoros, Atibaia, São Paulo, Brazil; 5 Programa de Pós-Graduação em Biodiversidade Tropical, Universidade Federal do Amapá, Macapá, Amapá, Brazil; Università degli Studi di Napoli Federico II, ITALY

## Abstract

Vertebrates are a vital ecological component of Amazon forest biodiversity. Although vertebrates are a functionally important part of various ecosystem services they continue to be threatened by anthropogenic impacts throughout the Amazon. Here we use a standardized, regularly spaced arrangement of camera traps within 25km^2^ to provide a baseline assessment of vertebrate species diversity in a sustainable use protected area in the eastern Brazilian Amazon. We examined seasonal differences in the per species encounter rates (number of photos per camera trap and number of cameras with photos). Generalized linear models (GLMs) were then used to examine the influence of five variables (altitude, canopy cover, basal area, distance to nearest river and distance to nearest large river) on the number of photos per species and on functional groups. GLMs were also used to examine the relationships between large predators [Jaguar (*Panthera onca*) and Puma (*Puma concolor*)] and their prey. A total of 649 independent photos of 25 species were obtained from 1,800 camera trap days (900 each during wet and dry seasons). Only ungulates and rodents showed significant seasonal differences in the number of photos per camera. The number of photos differed between seasons for only three species (*Mazama americana*, *Dasyprocta leporina* and *Myoprocta acouchy*) all of which were photographed more (3 to 10 fold increase) during the wet season. *Mazama americana* was the only species where a significant difference was found in occupancy, with more photos in more cameras during the wet season. For most groups and species variation in the number of photos per camera was only explained weakly by the GLMs (deviance explained ranging from 10.3 to 54.4%). Terrestrial birds (*Crax alector*, *Psophia crepitans* and *Tinamus major*) and rodents (*Cuniculus paca*, *Dasyprocta leporina* and *M*. *acouchy*) were the notable exceptions, with our GLMs significantly explaining variation in the distribution of all species (deviance explained ranging from 21.0 to 54.5%). The group and species GLMs showed some novel ecological information from this relatively pristine area. We found no association between large cats and their potential prey. We also found that rodent and bird species were more often recorded closer to streams. As hunters gain access via rivers this finding suggests that there is currently little anthropogenic impact on the species. Our findings provide a standardized baseline for comparison with other sites and with which planned management and extractive activities can be evaluated.

## Introduction

Currently, almost 37% of the Brazilian Amazon receives legal protection, with approximately 80.4% (~1.6 million km^2^) of the protected areas in Brazilian Amazonia allowing some form of human use [1]. The establishment of these protected areas was in many respects a world leading step to protect natural resources [2]. However the degradation of these efforts threatens both the conservation of biodiversity and human well-being [3]. Such degradation, combined with an uncertain future [4], means there is an urgent need to document the current state of biodiversity within the existing protected area networks [5].

Because the mechanisms that maintain biodiversity can differ with myriad factors including species interactions [6], the sensitivity of species to changes within and between landscapes [7, 8], and with their mobility within them [9], there is a need to understand the ecological factors affecting the distribution of different species to effectively manage and maintain biodiversity. For instance, large-bodied vertebrates are essential to maintain the structure and composition of tropical forests [10–12]. In the Guiana Shield and Central Amazonia frugivorous vertebrates alone disperse over 94% of all woody plant species [13]. For effective conservation it is key to study the ecology of these vertebrates [10, 14].

Despite their importance, there is lack of consistency in the methods used in studies on mid-sized and large bodied Amazon vertebrates [15, 16]. For example, numerous studies have used line transects and/or camera traps with different arrangements, lengths and sampling efforts [15, 17–19]. Such methodological differences make it difficult, if not impossible, to compare results across studies. Using a spatially standard sampling design that can be repeated across Amazonia is likely to improve the generation and communication of knowledge for the effective conservation and management of Amazon forests [20–22].

In this paper, we used a standardized sampling regime that has been utilized in several other tropical study sites to survey terrestrial vertebrates within a 25km^2^ area. Our study had four principal objectives: (1) to evaluate sampling effort and estimate species richness, (2) to test for differences between functional groups in their ecological relationships, (3) to test for differences between species in their ecological relationships, and (4) to compare the findings of our study to other similar studies in the neotropics and other tropical regions. Finally, we explore relevant considerations for management and conservation strategies in the Brazilian Amazon.

## Materials and Methods

### Ethics Statement

Data collection used non-invasive, remotely activated camera traps and did not involve direct contact or interaction with animals. Fieldwork was conducted under research permit number IBAMA/SISBIO 40355–1 to LJM, DN, and FM, issued by the Instituto Chico Mendes de Conservação da Biodiversidade (ICMBio).

### Study Area

**Fig 1:**
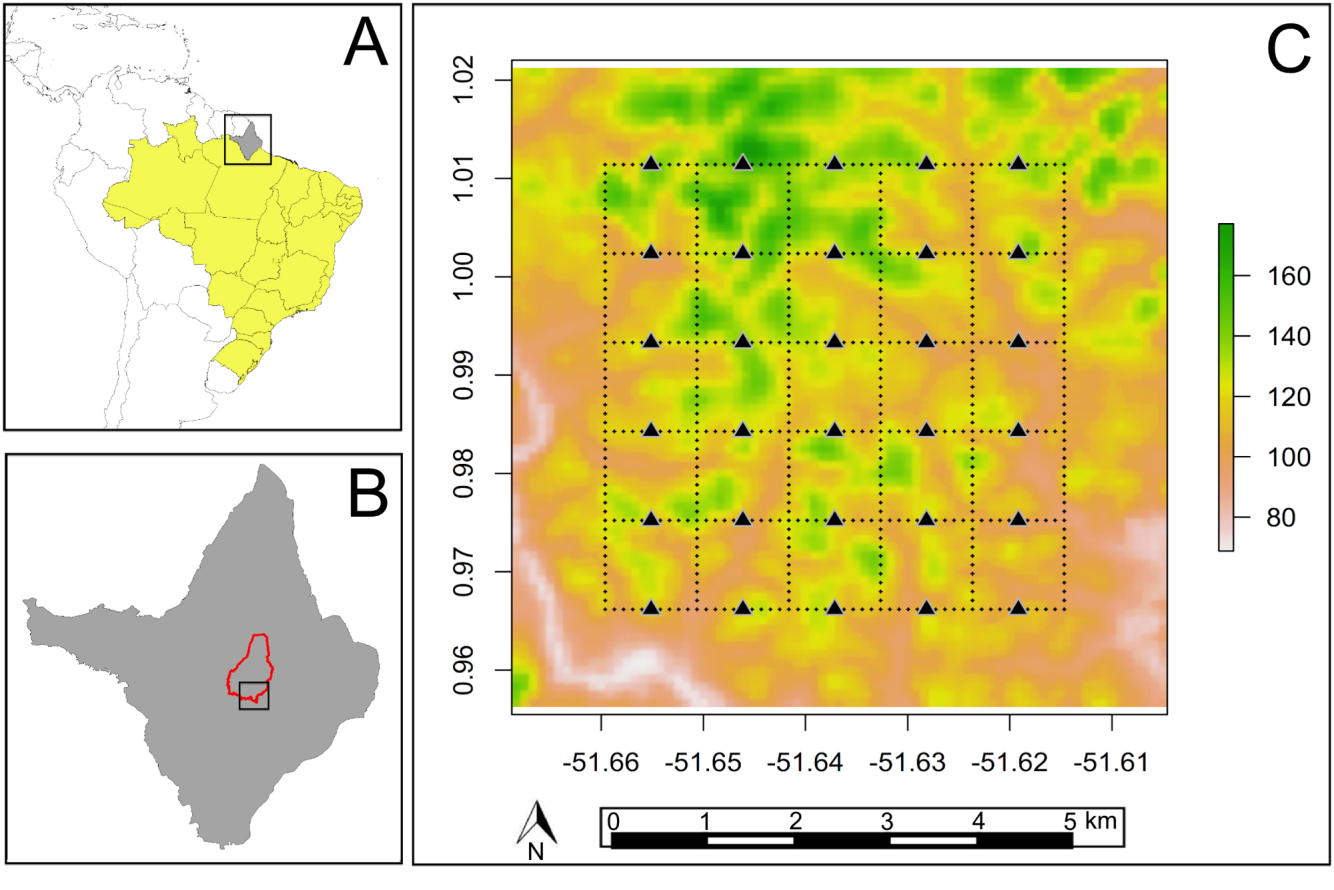
Location of the study region in the Amapá National Forest (ANF), Amapá State, eastern Brazilian Amazon. (A) Amapá State in Brazil; (B) ANF (red polygon) in Amapá State; (C) SRTM image showing altitude across the grid system (dotted lines) where the study was conducted. Camera traps were placed at 30 regularly spaced sample points (black triangles).

This study was conducted in Amapá National Forest (Floresta Nacional Amapá —hereafter ANF), a sustainable-use protected are of approximately 412,000 ha, located in the center of Amapá State in the extreme northeast of the Brazilian Amazon (0°55’29”N, 51°35’45”W, Fig 1) [23].

The ANF consists of continuous tropical rainforest vegetation, predominantly never-flooded “terra-firme” forest, with some areas of flooded forest, bamboo and rocky outcrops [24]. The ANF is part of a large (> 4 million hectares) connected group of protected areas (Fig 1, [23]) that maintain both continuous undisturbed forests and the complete regional community of medium-sized and large-bodied vertebrates. ANF currently experiences low levels of anthropogenic perturbation, in part because only eight families live on the reserve border, there are no major access roads and the nearest city is located 46 km away by river [25].

The regional climate is hot and humid, with annual rainfall ranging from 2,300 mm to 2,900 mm [26]. During the months with highest precipitation levels (February, March and April), rainfall may exceed 500 mm/month. The dry season (September to November) is characterized by a maximum precipitation below 250 mm/month [26].

### Sampling Design

Data were sampled in both dry (October to December 2013, with 182 mm cumulative precipitation) and wet (March to June 2014, with 789 mm cumulative precipitation) seasons. Data collection was conducted in a 25 km^2^ RAPELD grid (RAP surveys in the Long-term Ecological Research Sites whose Brazilian acronym is PELD, hence RAPELD) of the Brazilian Program for Biodiversity Research (PPBio) [20–22] (Fig 1). This standard grid consists of six north-south and six east-west 5 km trails. The current study used 30 regularly spaced sample points distributed at 1km intervals along the east-west trails (Fig 1, [20, 22]).

### Vertebrate Data

In order to sample vertebrates, we installed camera traps equipped with infrared triggers (Bushnell Trophy Cam, 8MP, Overland Park, KS, USA) in the RAPELD grid. As often reported from tropical systems (e.g. [27]), financial constraints meant we did not have sufficient cameras to survey the 30 points simultaneously. Cameras were therefore placed at 15 points for 30 consecutive days then immediately transferred to the remaining 15 points. All cameras were unbaited and installed 30–40 cm above the ground, facing the trail. Cameras functioned continuously (24 hours a day) during the 30-day sample period, which provided a sampling effort of 900 trap-days in each season. Cameras were programmed to film for 40 seconds post-activation, with intervals of 15 second between videos.

To estimate the relative abundance of vertebrates, we considered only independent videos, with over 30 min intervals in case of the same species recorded during the same day on the same camera [28, 29]. The species recorded by the camera traps were identified with the aid of standard field guides for regional mammals [30, 31] and birds [32, 33], with species identifications double-checked by 3 researchers each with more than 10 years regional experience (FM, TGO, and DN). Scientific names follow available checklists of mammals [34], and birds [35].

### Environmental Variables

To estimate the influence of environmental variables on vertebrates in each place where cameras were deployed, we measured the following variables: (i) canopy openness, (ii) number of trees, (iii) tree basal area, (iv) distance from the location of the cameras to the nearest large river, (v) distance from the location of the cameras to the nearest stream, and (vi) altitude.

Forest structure data (i.e., number of trees and basal area) were obtained from plots measuring 50 x 10 m, at the 30 points (at the same locations as camera traps). Canopy openness was quantified with a concave spherical canopy densiometer at five equidistant points within each plot. Four readings were taken per point [36]. The number of all trees ≥ 10 cm DBH (diameter at breast height at a standard 1.3 m above ground, or above tallest root buttress) was used to quantify the number of trees per area in each plot (m^2^). This count included all trees which had at least half of their basal trunk inside the plot.

Tree Basal Area in each plot was obtained as the sum of the basal area value for each individual tree derived from the DBH of each tree following the formula BA (basal area in m^2^/ha) = 0.00007854 X DBH^2^.

To estimate the altitude of the terrain at the camera location, we used a digital elevation model (DEM SRTM) produced by the Shuttle Radar Topographic Mission (SRTM) [37], with spatial resolution of 3 arc-second (approximately 90 m on the Equator), consisting of a set of elevations in digital format freely available on the internet (http://seamless.usgs.gov/ or http://www.cgiar-csi.org/data/srtm-90m-digital-elevation-database-v4-1). The geographical coordinates of the location of each camera trap were used to obtain the altitude of the terrain (DEM SRTM).

The distance from the camera traps to the nearest large river was estimated by using shapefiles of the Araguari and Falsino rivers (Fig 1, available at http://hidroweb.ana.gov.br/HidroWeb.asp?TocItem=4100), and measured as a straight line (Euclidian) distance with *Quantum Gis* version 2.4.0 [38].

Distances to the nearest river were derived from river locations within a GIS. This was done by using the SRTM DEM to generate river channel networks using standard GIS processes. We used SAGA (System for Automated Geoscientific Analyses) GIS ([39, 40], http://www.saga-gis.org/en/index.html), for data preprocessing and river channel network derivation (modules: “Fill Sinks (Wang & Liu)” and “Channel Network and Drainage Basins”).). We then calculated the straight-line distance from the location of each camera trap to the center of the nearest stream.

### Data Analysis

The relative abundance of each species was expressed as the number of independent videos per 10 trap-days [8, 41]. A Mann-Whitney U test was used to test for significant differences between the number of detections in the dry and rainy seasons, using a significance level of p < 0.05.

To assess whether the sampling effort in both seasons was sufficient to record the majority of species, we constructed and compared cumulative species curves with the *accumcomp* function of the *BiodiversityR* package [42]. To predict the total number of species that could be potentially detected in the area, we used four estimators (Chau, First order jackknife, Second order jackknife and Bootstrap), which extrapolate the species richness (i.e. estimate the number of undetected species) based on the frequency of recorded species (function *specpool*, package *Vegan*) [43].

To evaluate the correlation between the environmental variables, we examined pair-wise Spearman correlations between all variables. This preliminary analysis showed that there were no strong correlations (Spearman r < 0.70) between the environmental variables, with values ranging between 0.03 and 0.62, allowing all variables to be used in subsequent analyzes.

To test for differences in the ecological relationships of different functional groups and species we used Generalized Linear Models (GLMs, error distribution family = poisson). GLMs were preferred to alternatives such as occupancy models as the number of videos (i.e. potential recaptures) and naïve occupancy (proportion of cameras with records) was low for most species. For less common/rare species we can assume that differences in detectability were not affecting the GLM results [44]. To avoid overly complex models (total degrees of freedom in species GLMs = 30 points), preliminary variable selection [45] was used to select the five variables that showed higher weight of importance in the GLMs: canopy openness, basal area, distance to the nearest stream, distance to the largest river, and terrain altitude.

The GLMs were run separately for each species and for species divided into six functional groups. For the GLM analysis we selected only groups/species with at least one video in five or more different cameras within the study area. We defined the six functional groups as follows: (i) Birds (all birds), (ii) Large terrestrial Birds (Cracidae+Psophiidae), (iii) Ungulates (Artiodactyla+Perissodactyla), (iv) Large-bodied felids (*Puma concolor* + *Panthera onca)*, (v) Felids (all felids), and (vi) Rodents (all rodents). In the case of functional groups we also ran two additional models. To test for seasonal effects in each functional group the model consisted of the five variables mentioned above, plus the categorical variable ‘*season*’ with two levels (dry and rainy). Additionally we also used GLMs to examine the relationship between felids and potential prey species.

For individual species, we summed independent wet and dry season videos per camera. We then selected only those species with at least one video in five or more different cameras within the study area. All analyses were performed with the *R* language and environment for statistical computing [46].

## Results

### Sampling Effort and Species Richness

Following a sampling effort of 1800 trap-days (900 each for the dry and rainy seasons), we obtained 649 independent videos of 25 vertebrate species (Table 1). This total included four bird and 21 mammal species, representing 10 orders: Aves—Tinamiformes, Galliformes, Gruiformes; Mammals—Artiodactyla, Perissodactyla, Carnivora, Cingulata, Pilosa, Didelphimorphia and Rodentia (Table 1).

**Table 1:** Number of independent photos (Detection), number of cameras that recorded photos (NCP) and relative abundance in dry and wet seasons of all vertebrate species examined in this study.

Class / Order / Family	Species (Common name)	Detection^a^ (dry,wet)	NCP^b^ (dry,wet)	RA^c^ (dry,wet)
Birds				
Galliformes				
Cracidae	*Crax alector* (Black Curassow)	23 (9, 14)	13 (6, 9)	0.26 (0.1, 0.15)
Gruiformes				
Psophiidae	*Psophia crepitans* (Grey-winged Trumpeter)	110 (47, 63)	26 (17, 24)^†^	1.22 (0.52, 0.70)
Tinamiformes				
Tinamidae	*Crypturellus erythropus* (Red-legged Tinamou)	11 (11, 0)	1 (1, 0)	0.12 (0.12, 0)
	*Tinamus major* (Great Tinamou)	11 (4, 7)	6 (3, 3)	0.12 (0.04, 0.07)
Mammals				
Artiodactyla				
Cervidae	*Mazama americana* (Red Brocket Deer)	37 (6, 31)*	17 (4, 14)*	0.41 (0.06, 0.34)
	*Mazama nemorivaga* (Amazonian Brown Brocket Deer)	55 (36, 19)	25 (16, 14)	0.61 (0.4, 0.21)
Tayassuidae	*Pecari tajacu* (Collared Peccary)	77 (30, 47)	19 (13, 16)	0.86 (0.33, 0.52)
Perissodactyla				
Tapiridae	*Tapirus terrestris* (Lowland Tapir)	12 (5, 7)	8 (5, 7)	0.13 (0.05, 0.07)
Carnivora				
Felidae	*Leopardus pardalis* (Ocelot)	9 (1, 8)^†^	6 (1, 5)	0.10 (0.01, 0.08)
	*Leopardus wiedii* (Margay)	2 (1, 1)	2 (1, 1)	0.02 (0.01, 0.01)
	*Panthera onca* (Jaguar)	14 (7, 7)	12 (7, 6)	0.16 (0.07, 0.07)
	*Puma concolor* (Puma)	15 (5, 10)	10 (3, 8)	0.17 (0.05, 0.11)
Mustelidae	*Eira barbara* (Tayra)	7 (4, 3)	4 (3, 2)	0.08 (0.04, 0.03)
Procyonidae	*Nasua nasua* (South American Coati)	2 (0, 2)	2 (0, 2)	0.02 (0, 0.02)
	*Procyon cancrivorus* (Crab-eating Raccoon)	2 (0, 2)	1 (0, 1)	0.02 (0, 0.02)
Canidae	*Speothos venaticus* (Bush Dog)	1 (0, 1)	1 (0, 1)	0.01 (0, 0.01)
Cingulata				
Dasypodidae	*Dasypus kappleri* (Greater Long-nosed Armadillo)	8 (4, 4)	6 (3, 3)	0.09 (0.04, 0.04)
	*Dasypus novemcinctus* (Nine-banded Armadillo)	2 (2, 0)	2 (2, 0)	0.02 (0.02, 0)
Pilosa				
Myrmecophagidae	*Myrmecophaga tridactyla* (Giant Anteater)	7 (4, 3)	5 (4, 2)	0.08 (0.04, 0.03)
	*Tamandua tetradactyla* (Southern Tamandua)	2 (2, 0)	2 (2, 0)	0.02 (0.02, 0)
Didelphimorphia				
Didelphidae	*Didelphis marsupialis* (Black-eared Opossum)	3 (3, 0)	1 (1, 0)	0.03 (0.03, 0)
Rodentia				
Cuniculidae	*Cuniculus paca* (Spotted Paca)	18 (15, 3)	7 (5, 3)	0.20 (0.16, 0.03)
Dasyproctidae	*Dasyprocta leporina* (Red-rumped Agouti)	141 (32, 109)*	23 (16, 19)	1.57 (0.35, 1.21)
	*Myoprocta acouchy* (Red Acouchi)	77 (6, 71)*	13 (4, 10)	0.86 (0.06, 0.78)
Sciuridae	*Sciurus aestuans* (Guianan Squirrel)	3 (0, 3)	1 (0, 1)	0.03 (0, 0.03)

^a^ Number of detections with independent photos.

^b^ Number of cameras that recorded photos of the species.

^c^ Average relative abundance (number of independent photos per 10 camera-trap days).

* Differences between seasons. Mann-Whitney test: ^†^p <0.1, *p <0.05, **p<0.01, ***p<0.001.

**Fig 2:**
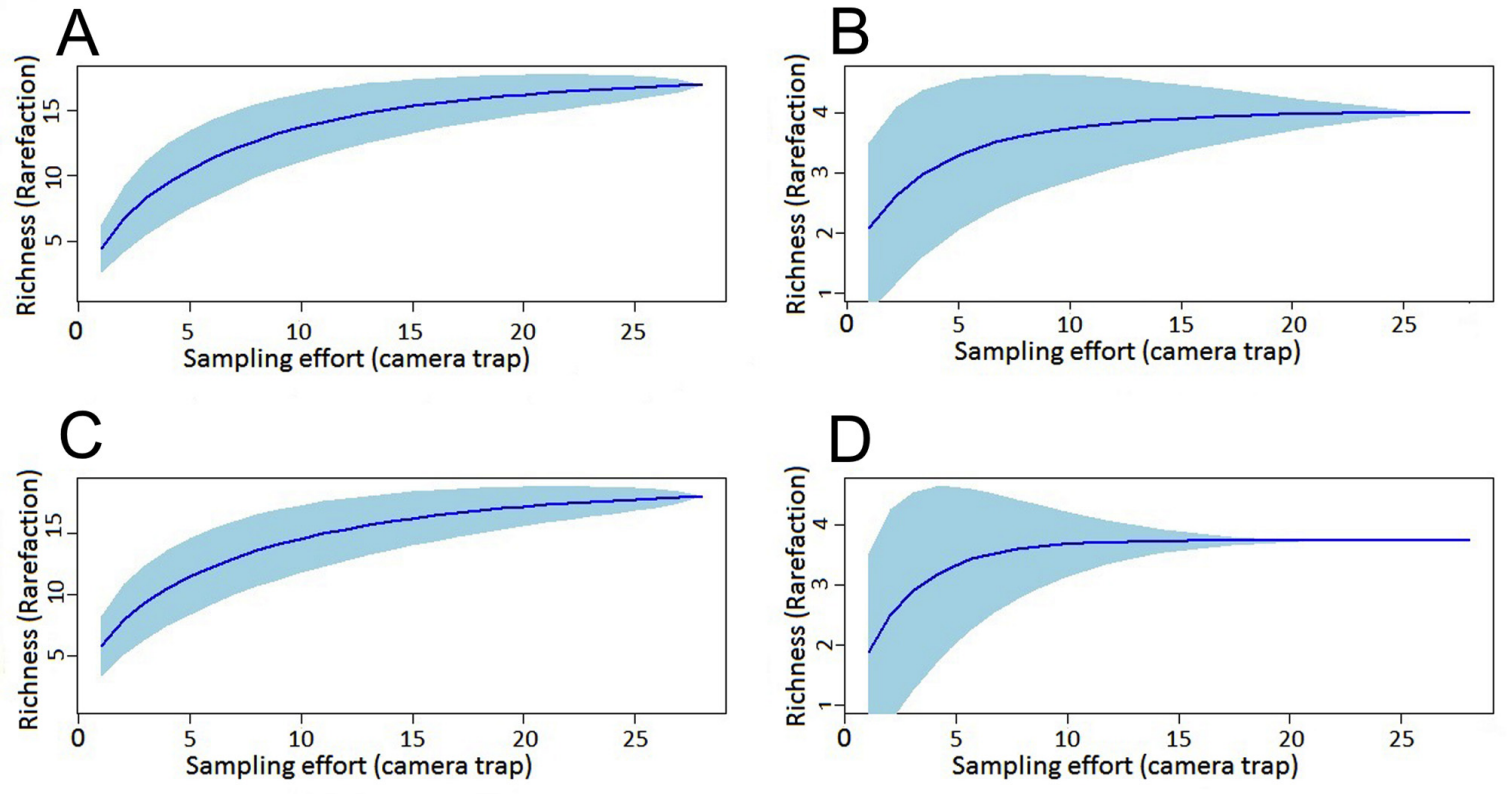
Cumulative curves for mammal and bird species sampled with camera traps in the dry and rainy seasons in the Amapá National Forest. Detection of species recorded in the 30 sample points is randomized 1000 times and results used to derive mean (dark blue line) 95% confidence intervals of the mean (light blue polygon). (A) Cumulative curve for mammal species in the dry season; (B) Cumulative curve for bird species in the dry season; (C) Cumulative curve for mammal species in the rainy season; (D) Cumulative curve for birds species in the rainy season.

The species accumulation curves show a tendency to stabilize (i.e. approached an asymptote) in both dry and rainy season samples, suggesting that sampling effort was sufficient for both mammals and birds (Fig 2). Comparison of the species richness estimates showed that we obtained between 84.0 and 91.4% of the species pool for mammals and 67.8 and 91.7% for birds (S1 Table).

**Fig 3:**
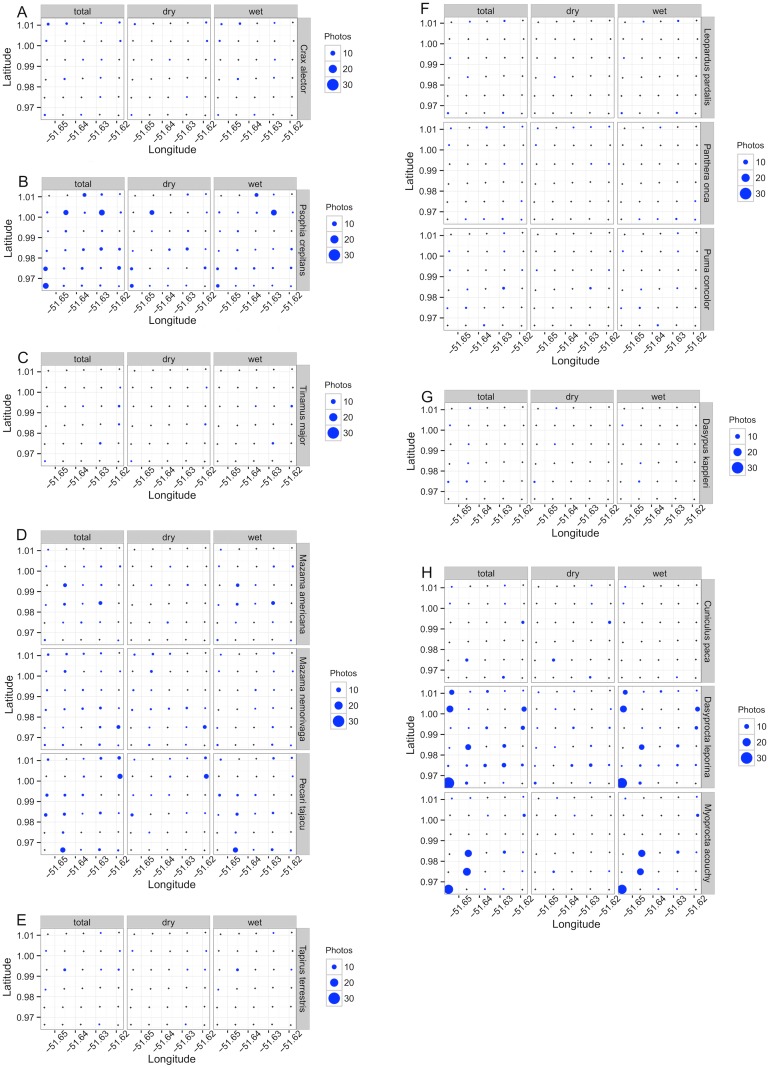
Number of photos per sampling point for vertebrate species sampled on a 25 km² grid, Amapá National Forest, Brazil. (A) Galliformes; (B) Gruiformes; (C) Tinamiformes; (D) Artiodactyla; (E) Perissodactyla; (F) Carnivora; (G) Cingulata; (H) Rodentia.

There were small differences between the species richness recorded in the wet and dry seasons (Fig 2, S1 Table). For mammals the observed and extrapolated richness increased (insignificantly) during the wet season (Fig 2, S1 Table). There were also seasonal differences in species composition (Fig 3A–3H, Table 1). Four species were recorded only in the dry season (*Crypturellus erythropus*, *Dasypus novemcinctus*, *Tamandua tetradactyla* and *Didelphis marsupialis*), and four exclusively in the rainy season (*Nasua nasua*, *Procyon cancrivorus*, *Speothos venaticus* and *Sciurus aestuans*).

The Mann-Whitney U test indicated that only three mammal species showed significant differences in the number of records (independent video records, Table 1) between the dry and rainy season sampling [*Mazama americana*, *Dasyprocta leporina* and *Myoprocta acouchy* (p = 0.023, 0.025 and 0.024 respectively)]. *Mazama americana* was the only species to show a difference between seasons in the number of cameras that recorded images (Table 1, p = 0.011).

We obtained an overall capture rate of 0.36 photos per trap-day (648 independent videos/1800 trap-days). *Dasyprocta leporina* had the highest relative abundance with 141 records (1.57 records/10 trap-days), followed by *Psophia crepitans* with 110 records (1.22 records/10 trap-days), and *Myoprocta acouchy* and *Tajacu peccari*, both with 77 records (0.86 records/10 trap-days) (Table 1).

### Functional Groups

The Generalized Linear Models (GLM's) indicated that the explanatory power of the model was low for almost all groups (Table 2), with a maximum deviance explained of 40% (for rodents) and a minimum of 10% (for birds). The group representing total summed bird abundance (*All birds*) was negatively influenced by the variables *canopy openness*, *distance to major river* and *distance to nearest stream*. The avian group containing only large terrestrial birds (Cracidae and Psophiidae) was negatively influenced by *canopy openness*, *distance to major river* and *distance to nearest stream*, and was positively influenced by *tree basal area*. The group *Ungulates* was positively influenced only by the variable *distance to the nearest stream*. The two groups of felids were negatively influenced by *canopy openness* and *altitude*, while the group representing all felid records (*All felids*) was also positively influenced by *tree basal area* (S2 Table). The two prey categories (*Prey < 5kg* and *Prey > 5kg*) did not significantly explain variation in the felid groups (S3 Table). *Rodents* were the group with the greatest number of significant variables, showing negative associations with *canopy openness*, *distance to major river* and *distance to nearest stream*, while *altitude* was positively associated with relative abundance in this group (Table 2).

**Table 2:** Parameter (Slope) estimates of explanatory variables from the GLMs on the abundance of groups of vertebrates in the eastern Brazilian Amazon.

Groups	Canopy Openness		Altitude		Basal area		Distance to large rivers		Distance to stream		Model	
	Slope (SE)^a^	Z value	Slope (SE)^a^	Z value	Slope (SE)^a^	Z value	Slope (SE)^a^	Z value	Slope (SE)^a^	Z value	DE (%)^b^	AIC^c^
All birds	-0.174 (0.078)	-2.21*	0.002 (0.003)	0.71^†^	0.095 (0.067)	1.40^†^	-0.203 (0.093)	-2.18*	-0.000 (0.000)	-1.76^†^	15.27	190.39*
Birds (Cracidae + Psophiidae)	-0.186 (0.089)	-2.09*	0.020 (0.010)	1.95^†^	0.190 (0.084)	2.25*	-0.169 (0.112)	-1.51^†^	-0.001 (0.000)	-2.26*	11.90	266.70**
Ungulates^d^	0.071 (0.067)	1.05^†^	-0.006 (0.003)	-1.79^†^	-0.012 (0.072)	-0.16^†^	-0.025 (0.082)	-0.31^†^	0.000 (0.000)	2.61**	11.79	185.91^†^
Large bodied felids^e^	-0.476 (0.242)	-1.96*	-0.079 (0.035)	-2.25*	0.146 (0.148)	0.98^†^	0.276 (0.223)	1.23^†^	0.002 (0.001)	1.61^†^	23.41	110.59*
All felids	-0.239 (0.161)	-1.48^†^	-0.019 (0.008)	-2.45*	0.305 (0.131)	2.32*	0.001 (0.175)	0.00^†^	0.000 (0.000)	0.85^†^	29.59	91.68*
All rodents	-0.034 (0.077)	-0.44^†^	-0.029 (0.008)	3.39***	0.092 (0.072)	1.27^†^	-0.846 (0.103)	-8.20***	-0.002 (0.000)	-8.71***	39.78	445.62***

Significance values: ^†^not significant, *p <0.05, **p<0.01, ***p<0.001.

^a^ Slope for variables and Standard Error (SE);

^b^ Percentage of Deviance Explained for each model (DE (%));

^c^ Akaike Information Criterion value for each model (AIC);

^d^ Includes all Artiodactyla and Perissodactyla recorded in the study area.

^e^ Includes only large-bodied felids (*Puma concolor* and *Panthera onca*).

### Species

Of the 14 species assessed in the GLMs, seven showed statistically significant results (Table 3).

However, the percentage variation explained by the model was low for almost all species, ranging from a minimum of 16% for *Tajacu peccari* to a maximum of 54% for *Cuniculus paca*. Of these 14 species, the birds *C*. *alector*, *P*. *crepitans* and *T*. *major*, and rodents *C*. *paca*, *D*. *leporina* and *M*. *acouchy* were the species where the model provided the highest percentage of explanation for their distributions, ranging from 21.0 to 54.5%.

The species with the greatest number of significant variables in the model was *M*. *acouchy* (four variables), followed by *C*. *paca*, *D*. *leporina* and *P*. *crepitans*, all with three significant variables. Four species (*Mazama nemorivaga*, *Leopardus pardalis*, *Panthera onca* and *Dasypus kappleri*) were not associated significantly with any of the environmental variables in the model (Table 3).

**Table 3:** Parameter (Slope) estimates from GLMs analysis of the abundance of vertebrate species in the eastern Brazilian Amazon.

Class / Family / Species	Canopy Openness		Altitude		Basal area		Distance to large rivers		Distance to stream		Model	
	Slope (SE)^a^	Z value	Slope (SE)^a^	Z value	Slope (SE)^a^	Z value	Slope (SE)^a^	Z value	Slope (SE)^a^	Z value	DE (%)^b^	AIC^c^
Birds												
Cracidae												
*Crax alector*	-0.435 (0.219)	-1.98*	-0.004 (0.009)	-0.43^†^	-0.627 (0.307)	-2.04*	0.288 (0.267)	1.08^†^	0.001 (0.001)	1.70^†^	23.87	76.64***
Psophiidae												
*Psophia crepitans*	-0.233 (0.093)	-2.48*	-0.008 (0.004)	1.74^†^	0.169 (0.074)	2.28*	-0.170 (0.111)	-1.52^†^	-0.001 (0.000)	-2.53*	21.21	167.43**
Tinamidae												
* Tinamus major*	0.871 (0.276)	3.15**	0.013 (0.016)	0.80^†^	-0.239 (0.300)	-0.79^†^	-0.003 (0.452)	-0.00^†^	-0.003 (0.002)	-1.55^†^	42.99	48.27**
Mammals												
Cervidae												
* Mazama americana*	0.053 (0.156)	0.34^†^	-0.020 (0.007)	-2.71**	-0.108 (0.179)	-0.60^†^	0.285 (0.183)	1.56^†^	0.001 (0.000)	2.06*	15.83	107.75^†^
* Mazama nemorivaga*	0.052 (0.118)	0.44^†^	0.006 (0.006)	1.01^†^	0.166 (0.104)	1.60^†^	-0.026 (0.153)	-0.17^†^	-0.000 (0.000)	-0.86^†^	12.05	109.53^†^
Tayassuidae												
*Pecari tajacu*	0.040 (0.107)	0.37^†^	-0.003 (0.005)	-0.63^†^	-0.209 (0.137)	-1.52^†^	-0.171 (0.131)	-1.30^†^	0.001 (0.000)	2.96**	16.19	158.23**
Tapiridae												
* Tapirus terrestris*	0.315 (0.293)	1.07^†^	-0.033 (0.014)	-2.30*	0.065 (0.303)	0.21^†^	-0.101 (0.327)	-0.31^†^	0.002 (0.001)	1.46^†^	19.54	58.72^†^
Felidae												
* Leopardus pardalis*	-0.850 (0.459)	-1,84^†^	-0.027 (0.010)	-1.55^†^	-0.090 (0.425)	-0.21^†^	-0.069 (0.408)	-0.17^†^	0.001 (0.001)	0.85^†^	26.79	47.79^†^
* Panthera onca*	-0.562 (0.294)	-1.91^†^	-0.024 (0.014)	-1.75^†^	0.284 (0.238)	1.19^†^	0.187 (0.301)	0.62^†^	0.000 (0.001)	-0.08^†^	29.55	56.16^†^
* Puma concolor*	0.057 (0.245)	0.23^†^	-0.015 (0.014)	-1.07^†^	0.519 (0.187)	2.77**	0.002 (0.290)	0.00^†^	0.001 (0.001)	0.83^†^	20.87	64.11^†^
Dasypodidae												
* Dasypus kappleri*	-0.655 (0.474)	-1.38^†^	0.011 (0.018)	0.61^†^	0.071 (0.373)	0.19^†^	-0.790 (0.458)	-1.72^†^	-0.000 (0.002)	-0.34^†^	26.69	45.74^†^
Cuniculidae												
* Cuniculus paca*	-0.080 (0.318)	-0.25^†^	-0.032 (0.013)	-2.33*	0.397 (0.184)	2.15*	-0.703 (0.379)	-1.85^†^	-0.006 (0.002)	-2.57**	54.51	58.28**
Dasyproctidae												
* Dasyprocta leporina*	0.007 (0.087)	0.91*	0.000 (0.004)	0.11^†^	-0.123 (0.084)	-1.45^†^	-0.000 (0.000)	-5.03***	-0.001 (0.000)	-2.95**	21.00	250.13***
* Myoprocta acouchy*	-0.364 (0.154)	-2.35*	-0.134 (0.666)	-2.35*	0.213 (0.102)	2.09^†^	-0.848 (0.154)	-5.48***	-0.001 (0.000)	-2.29*	31.06	214.52***

Significance values: ^†^not significant, *p <0.05, **p<0.01, ***p<0.001.

^a^ Slope for variables and Standard Error (SE);

^b^ Percentage of Deviance Explained for each model (DE (%));

^c^ Akaike Information Criterion value for each model (AIC).

The variable *canopy openness* had a negative influence on abundance in *Crax alector*, *Psophia crepitans* and *Myoprocta acouchy*. This same variable positively influenced abundance in *Dasyprocta leporina* and *Tinamus major*. The variable *altitude* negatively influenced the abundance of *Mazama americana*, *Tapirus terrestris*, *Cuniculus paca* and *Myoprocta acouchy*. *Tree basal area* negatively influenced abundance in *Crax alector*, and positively influenced abundance in *Psophia crepitans*, *Puma concolor* and *Cuniculus paca*. *Distance to nearest large river* influenced negatively *Dasyprocta leporina* and *Myoprocta acouchy* abundance. Finally, *distance to nearest stream* negatively affected abundance in *Psophia crepitans*, *Cuniculus paca*, *Dasyprocta leporina* and *Myoprocta acouchy*, while positively affected those of *Mazama americana* and *Pecari tajacu* (Table 3).

## Discussion

Our analysis of medium and large vertebrates in a 25 km^2^ area of lowland tropical forest showed that, although overall the model explained little of the species composition in the area, the sampled environmental variables themselves are important for species composition, with meso-scale variations in forest structure, topography and watercourse proximity significantly influencing species occurrence. Linking presence and abundance to the ecological requirements of vertebrates in question, such fine-tuned ecological knowledge is fundamental to the effective conservation of these species [47, 48].

### Sampling Effort and Species Richness

The differences between the observed and extrapolated species richness values obtained for birds and mammals combined indicates that we recorded between 80 and 90% of the species in the study area. This finding suggests that our sampling effort was sufficient to capture most of the species and that our results are suitable for within and between site comparisons.

We recorded the full range of terrestrial medium to large bodied mammals (from agoutis to jaguars), which was to be expected considering the remote location of our study area. Indeed, the 21 medium and large bodied mammal species recorded by our study is a similar number to that recorded for other Amazonian regions [49–51], and for other areas in the State of Amapá [52]. However, we did not detect some species that have been widely recorded across the Guiana Shield, such as *Tayassu pecari*, *Priodontes maximus*, and *Puma yagouaroundi* [53]. Although thought to be relatively rare across Amazonia, these three species were recorded for the ANF in a rapid biological inventory [54].

The fact that we did not record some species is to be expected as many mammal species are difficult to detect and have relatively large home ranges, hence may require greater sampling effort [51] and/or the use of complementary techniques [8, 55]. The rapid inventory [54] was based on a smaller sampling effort (20 days of fieldwork with 62 hours of active search), but used a combination of indirect and direct techniques. These techniques included five camera traps distributed in front of dens and places with signs of vertebrate activity. Additionally the camera traps were baited with honey, bacon, carrot and orange [54]. Thus, the reason for not recording some species that occur in our study area could be related with the use of only one method, as the use of complementary techniques have been proven to be more efficient for surveying vertebrates than single methods [55]. It is also possible that the sampling effort was not sufficient to detect locally rare species that have been recorded with camera traps elsewhere in Amazonia [51, 56]. Also, some species that were not detected, such as *T*. *pecari* are known to range widely [57] and follow seasonal changes in habitat and resource availability [58], which are both characteristics that could make it difficult to detect these species. Other non-detected species such as the *Puma yagouaroundi*, are also rare in the Amazon [51] and more associated with open habitats [59], although also occurring in dense forest cover [60]. Thus, *P*. *yagouaroundi* may not be so easily detected in core pristine forest areas.

We recorded four large terrestrial bird species, a much lower number than that described for the ANF in a rapid biological inventory [61]. This inventory was based on a combination of mist-nets and sound records and identified nine large bird species (Tinamidae, Cracidae and Psophiidae). All nine species are likely to be recorded by camera traps due to their large body size and habit of foraging on the ground. Thus (as suggested by our extrapolated bird richness values), we registered approximately half of the bird species that could possibly be recorded with terrestrial camera traps in the study area. Nevertheless, other camera trap studies [51], using more than double our survey effort over two years, also recorded a similarly low richness (4 species) of large ground-dwelling birds. This low richness suggests that this technique might not be ideal for this group of birds. For example, only one large terrestrial bird (*Crax alector*) was recorded during 459 camera trap/days in a study conducted in ANF to monitor latrines of giant otters (*Pteronura brasiliensis*) [62]. The fact that some bird species were photographed only in the wet season and others only in the dry season is also likely a reflection of sampling effort, as there is no other plausible explanation given their known ecological features [30].

### Differences between functional groups

Ungulates and rodents were the groups most strongly influenced by season, with greater numbers of records in the rainy season. Such differences may be associated with between-season fluctuations in the level of resource availability, and may not be due to an increase in the number of individuals but by an increase in the number of times resident animals are recorded as they increase their activities in the area when local resources abound [19]. These seasonal differences agree with another study in the Amazon with medium and large-bodied vertebrates [49], but are contrary to the results of another in the same biome with medium and large mammals [63], which had more records in the dry season. We suggest that future studies aiming to evaluate abundance and occupancy rates of medium and large terrestrial mammals and birds in the tropics include rainy season sampling due to such seasonal differences.

Many camera trap studies [64] are often conducted during the dry season (months with less than 100 mm average rainfall) due to logistical constraints associated with rainy season surveys (e.g. restricted access and cameras malfunctioning). Due to such logistical constraints we still know very little regarding patterns in Amazon biodiversity during the rainy season. If camera traps were associated with phenological and resource availability studies (much of Amazon fruit production occurs during the rainy season [65–67]), it may also be possible to better understand the processes driving the spatial and temporal distribution of these species.

*Birds* and *Rodents* were negatively influenced by the variables *distance to nearest stream* and *distance to nearest large river*, showing an increase in the number of records closer to water bodies. This finding was not unexpected as the preference of this group of birds for moister areas and the preference of rodents (particularly paca) for areas close to water has been documented previously [68–72]. Although rivers are the main means of human transport in the region and these groups are hunted by local inhabitants [25, 73, 74], they were still recorded close to the large rivers, which suggests that there is little impact of humans within the ANF.

Records of Galliformes, Gruiformes and Tinamiformes (*Birds*) were also positively associated with areas of denser canopy and with areas of denser ground vegetation (greater tree basal area), corroborating studies that found these ground dwelling birds to exhibit this preference in such habitats [68].

Large felids were associated with lowland areas and areas of denser vegetation, but unexpectedly showed no association with prey (variables *Prey <5kg* and *Prey> 5kg*). Although other studies have shown prey availability to influence the distribution of predators in rainforest habitats [75], this could not be detected at the ANF, perhaps due to the broad distribution througout the area of both predators and prey. It seems likely that with such a variety and number of potential prey species these predators are not limited by prey availability.

### Differences between species

Overall, our capture rate was similar to those reported from other protected Amazon forests (51), and greater than those reported from fragmented/more disturbed/less productive Neotropical sites (see table 2 in [76]). The species relative abundances detected at the ANF were also similar to those found using camera-traps in other Amazon sites [51, 56]. Of the most commonly recorded species, *Dasyprocta leporina* and *M*. *acouchy* showed a clear preference for low-lying areas near large rivers, a pattern well-known from previous studies of these rodents [69, 71, 72]. In contrast the distributions of three bird species appeared to be most closely related to the variables describing forest structure [47]. *Crax alector* and *P*. *crepitans* were more frequently recorded in closed canopy areas with greater forest cover, while *T*. *major* was most often recorded from more open areas. This may be a result of behaviors associated mainly with ground foraging [68].

The ungulates and the two big cats (*P*. *concolor* and *P*. *onca*) did not appear to be greatly influenced by the sampled variables. Both groups comprise wide-ranging species with non-specialized habits [30, 77]. It therefore appears that these species have enough ecological/behavioral plasticity for them not to be strongly affected/limited by the measured variables on a meso-scale. This lack of association with the environmental variables examined suggests that other factors such as biotic interactions and resource availability [78–80] maybe more important determinants of species distributions and densities in the ANF.

We must of course remain cautious in our conclusions. While capture frequencies can give an idea of the relative abundance of different species, there is an ongoing discussion among scientists about the reliability of this index [81, 82] and how such indexes relate to population parameters. For example, results from within any area may be affected by individual or species specific factors such as trail use or avoidance, vertical and horizontal space use (e.g. partly arboreal versus exclusively terrestrial), or habitat specialist versus generalist. For this reason we limit our conclusions to differences in encounter rates and do not attempt to imply population parameters (e.g. density).

It is important to remember that the synergistic impacts of anthropogenic disturbances such as logging and hunting often decimate species richness and populations of Amazon vertebrates [8, 73, 77]. These impacts are not subtle and are orders of magnitude beyond documented errors/uncertainties in camera trap surveys [82]. Despite such uncertainties, we believe that a combination of a standardized survey and remotely triggered camera traps means that findings are comparable between sites using similar spatially standardized sample arrangements (e.g. [16, 27, 51, 56, 64]). Additionally, our findings also serve as a robust baseline for monitoring changes in species encounters and composition in response to the extractive activities proposed in the ANF management plan [22, 83].

## Conclusions

The inability of the causal model to explain the distribution of the recorded species suggests that, at the meso-scale level (25 km^2^), environmental variables had little influence on the relative abundance, richness and species distribution of medium and large terrestrial vertebrates at our study area. Consequently, other factors may have more decisive roles at this scale, such as biotic interactions between species and the availability of resources [78–80]. Our findings suggest environmental integrity within the protected area, and also indicate that there is currently little human disturbance. Continued monitoring is required to ensure that proposed extractive activities do not disrupt the apparently intact community or its ecological interactions.

## Supporting Information

S1 Table Observed and extrapolated species richness. (DOCX)

S2 Table Parameter (Slope) estimates of explanatory variables (adding seasonality) from the GLMs on the abundance of groups of vertebrates in the eastern Brazilian Amazon. (DOCX)

S3 Table Parameter (Slope) estimates of prey variables from the GLMs on the abundance of felid groups in the eastern Brazilian Amazon. (DOCX)
