# Supplementary material for: Ecological Relationships of Meso-Scale Distribution in 25 Neotropical Vertebrate Species
Source: PLoS One. 2015 May 4;10(5):e0126114. doi: 10.1371/journal.pone.0126114 (PMC4418742; doi:10.1371/journal.pone.0126114)
Supplement: S1 Table — (DOCX) [file pone.0126114.s001.docx]

**S1 Table. Observed and extrapolated species richness.**

Species richness of medium to large bodied mammals and birds sampled with camera traps in the dry and rainy seasons in the Amapá National Forest. Extrapolations based on four estimators with standard errors (“SE”) in parenthesis.

|  | | | | Observed | Extrapolated richness estimates^a^ | | | |
| --- | --- | --- | --- | --- | --- | --- | --- | --- |
|  |  |  |  |  | Chau (SE) | First order jackknife (SE) | Second order jackknife ^b^ | Bootstrap (SE) |
| **All** | | |  | **25** | **28.1 (3.7)** | **29.8 (2.2)** | **30.9** | **27.3 (1.3)** |
|  | Dry | | | 21 | 25.0 (5.3) | 24.9 (1.9) | 26.8 | 22.9 (1.2) |
|  | Rainy | | | 21 | 23.7 (3.5) | 24.9 (1.9) | 25.9 | 23.0 (1.2) |
| **Mammals** | | |  | **21** | **23.0 (2.6)** | **24.9 (1.9)** | **25.0** | **23.0 (1.3)** |
|  | Dry | | | 17 | 19.3 (3.4) | 19.9 (1.7) | 20.9 | 18.5 (1.1) |
|  | Rainy | | | 18 | 20.7 (3.5) | 21.9 (1.9) | 22.9 | 19.9 (1.2) |
|  |  | | |  |  |  |  |  |
| **Birds** | |  | | **4** | **4.0 (0.0)** | **5.0 (1.0)** | **5.9** | **4.4 (0.5)** |
|  | Dry | | | 4 | 4.0 (0.0) | 5.0 (1.0) | 5.9 | 4.4 (0.6) |
|  | Rainy | | | 3 | 3.0 (0.0) | 3.0 (1.0) | 3.0 | 3.0 (0.2) |
|  |  | | |  |  |  |  |  |

^a^ Extrapolations calculated using incidence-based estimates i.e. the frequencies of species in the collection of 30 sample points. Four different variants were used to estimate the extrapolated species richness in the species pool.

^b^ Variance estimator not yet implemented.
