# Supplementary material for: Ecological Relationships of Meso-Scale Distribution in 25 Neotropical Vertebrate Species
Source: PLoS One. 2015 May 4;10(5):e0126114. doi: 10.1371/journal.pone.0126114 (PMC4418742; doi:10.1371/journal.pone.0126114)
Supplement: S2 Table — (DOCX) [file pone.0126114.s002.docx]

**S2 Table. Parameter (Slope) estimates of explanatory variables (adding seasonality) from the GLMs on the abundance of groups of vertebrates in the eastern Brazilian Amazon.**

| Groups | Canopy Openness | | Altitude | | Basal area | | Distance to large rivers | | Distance to stream | | Season | | Model | |
| --- | --- | --- | --- | --- | --- | --- | --- | --- | --- | --- | --- | --- | --- | --- |
|  | Slope  (SE) | Z  value | Slope  (SE) | Z  value | Slope  (SE) | Z  value | Slope  (SE) | Z  value | Slope  (SE) | Z  value | Slope  (SE) | Z  value | DE (%) | AIC |
| All birds | -0.121  (0.082) | -1.51^†^ | 0.019  (0.009) | 1.91^†^ | 0.169  (0.078) | 2,16* | -0.281  (0.105) | -2.67** | -0.001  (0.000) | -2.46* | 0.168  (0.161) | 1.04^†^ | 10.33 | 310.4** |
| Birds (Cracidae + Psophiidae) | -0.186  (0.089) | -2.09* | 0.020  (0.010) | 1.95^†^ | 0.190  (0.084) | 2.25* | -0.169  (0.112) | -1.51^†^ | -0.001  (0.000) | -2.26* | 0.318  (0.175) | 1.81^†^ | 14.15 | 265.3** |
| Ungulates^a^ | 0.078  (0.072) | 1.08^†^ | 0.001  (0.009) | 0.11^†^ | -0.010  (0.080) | -0.11^†^ | -0.066  (0.089) | -0.74^†^ | 0.000  (0.000) | 1.47^†^ | 0.300  (0.150) | 1.99* | 11.79 | 319.02^†^ |
| Large-bodied felids^b^ | -0.476  (0.242) | -1.96* | -0.079  (0.035) | -2.25* | 0.146  (0.148) | 0.98^†^ | 0.276  (0.223) | 1.23^†^ | 0.002  (0.001) | 1.61^†^ | 0.348  (0.377) | 0.92^†^ | 23.41 | 110.59* |
| All felids | -0.425  (0.194) | -2.18* | -0.062  (0.027) | -2.31* | 0.068  (0.137) | 0.49^†^ | 0.142  (0.191) | 0.74^†^ | 0.001  (0.001) | 0.65^†^ | 0.619  (0.331) | 1.86^†^ | 21.12 | 133.38* |
| All rodents | -0.034  (0.077) | -0.44^†^ | -0.029  (0.008) | 3.39*** | 0.092  (0.072) | 1.27^†^ | -0.846  (0.103) | -8.20*** | -0.002  (0.000) | -8.71*** | 1.555  (0.178) | 8.71*** | 39.78 | 445.62*** |

Slope for variables and Standard Error (SE); Z value for variables; Percentage of Deviance Explained for each model (DE (%)); Akaike Information Criterion value for each model (AIC); Significance: ^†^not significant, *p <0.05, **p<0.01, ***p<0.001.

^a^ Includes all Artiodactyla and Perissodactyla.

^b^ Includes only *Puma concolor* and *Panthera onca*.
