# Supplementary material for: Ecological Relationships of Meso-Scale Distribution in 25 Neotropical Vertebrate Species
Source: PLoS One. 2015 May 4;10(5):e0126114. doi: 10.1371/journal.pone.0126114 (PMC4418742; doi:10.1371/journal.pone.0126114)
Supplement: S3 Table — (DOCX) [file pone.0126114.s003.docx]

**S3 Table. Parameter (Slope) estimates of prey variables from the GLMs on the abundance of felid groups in the eastern Brazilian Amazon.**

| Groups | Canopy Openness | | Altitude | | Basal area | | Distance to large rivers | | Distance to stream | | Prey <5kg | | Prey >5kg | | Model | |
| --- | --- | --- | --- | --- | --- | --- | --- | --- | --- | --- | --- | --- | --- | --- | --- | --- |
|  | Slope  (SE) | Z  value | Slope  (SE) | Z  value | Slope  (SE) | Z  value | Slope  (SE) | Z  value | Slope  (SE) | Z  value | Slope  (SE) | Z  value | Slope  (SE) | Z  value | DE (%) | AIC |
| Large bodied felids^a^ | -0.495 (0.249) | -1.98* | -0.078 (0.036) | -2.19* | 0.142 (0.151) | 0.93^†^ | 0.274 (0.235) | 1.16^†^ | 0.002 (0.001) | 1.54^†^ | 0.291 (0.398) | 0.73^†^ | 0.004 (0.024) | 0.17^†^ | 24.95 | 113.61* |
| All felids | -0.449 (0.203) | -2.20* | -0.064 (0.028) | -2.28* | 0.066 (0.142) | 0.46^†^ | 0.186 (0.203) | 0.91^†^ | 0.002 (0.001) | 1.68^†^ | 0.490 (0.352) | 1.39^†^ | 0.013 (0.016) | -0.78^†^ | 24.1 | 135.16* |

Slope for variables and Standard Error (SE); Z value for variables; Percentage of Deviance Explained for each model (DE (%)); Akaike Information Criterion value for each model (AIC); Significance values: ^†^not significant, *p <0.05.

^a^ Includes only *Puma concolor* and *Panthera onca*.
